# Supplementary material for: Industry ties and evidence in public comments on the FDA framework for modifications to artificial intelligence/machine learning-based medical devices: a cross sectional study
Source: BMJ Open. 2020 Oct 14;10(10):e039969. doi: 10.1136/bmjopen-2020-039969 (PMC7559037; doi:10.1136/bmjopen-2020-039969)
Supplement: Reviewer comments [file bmjopen-2020-039969.reviewer_comments.pdf]

PEER REVIEW HISTORY

BMJ Open publishes all reviews undertaken for accepted manuscripts. Reviewers are asked to complete a checklist review form (**http://bmjopen.bmj.com/site/about/resources/checklist.pdf**) and are provided with free text boxes to elaborate on their assessment. These free text comments are reproduced below.

ARTICLE DETAILS

**TITLE (PROVISIONAL)**	Industry ties and evidence in public comments on the FDA framework for modifications to artificial intelligence/machine learning-based medical devices: a cross sectional study
**AUTHORS**	Smith, James; Abhari, Roxanna; Hussain, Zain; Heneghan, Carl; Collins, Gary; Carr, Andrew

This article was not externally reviewed at BMJ Open. Protocols that have been independently assessed prior to submission to BMJ Open are usually fast-tracked to publication on the grounds that further substantial changes will not be possible. This independent assessment will usually be external, independent review for both a substantial grant award from a non-commercial or government funder and ethics approval.
